# Targeted On-Demand Screening of Pesticide Panel in Soil Runoff

**DOI:** 10.3389/fchem.2021.782252

**Published:** 2021-11-30

**Authors:** Vikram Narayanan Dhamu, Suhashine Sukumar, Crisvin Sajee Kadambathil, Sriram Muthukumar, Shalini Prasad

**Affiliations:** ^1^ Department of Bioengineering, Biomedical Microdevices and Nanotechnology Laboratory, University of Texas at Dallas, TX, Richardson, United States; ^2^ EnLiSense LLC, TX, Allen, United States

**Keywords:** field-deployable sensing platform, soil runoff sensor, pesticide screening, soil pollution analysis, environmental sensor, impedimetric biosensor

## Abstract

Using pesticides is a common agricultural and horticultural practice to serve as a control against weeds, fungi, and insects in plant systems. The application of these chemical agents is usually by spraying them on the crop or plant. However, this methodology is not highly directional, and so only a fraction of the pesticide actually adsorbs onto the plant, and the rest seeps through into the soil base contaminating its composition and eventually leaching into groundwater sources. Electrochemical sensors which are more practical for *in situ* analysis used for pesticide detection in soil runoff systems are still in dearth, while the ones published in the literature are attributed with complex sensor modification/functionalization and preprocessing of samples. Hence, in this work, we present a highly intuitive electroanalytical sensor approach toward rapid (10 min), on-demand screening of commonly used pesticides—glyphosate and atrazine—in soil runoff. The proposed sensor functions based on the affinity biosensing mechanism driven *via* thiol cross-linker and antibody receptors that holistically behaves as a recognition immunoassay stack that is specific and sensitive to track test pesticide analytes. Then, this developed sensor is integrated further to create a pesticide-sensing ecosystem using a front-end field-deployable smart device. The method put forward in this work is compared and validated against a standard laboratory potentiostat instrument to determine efficacy, feasibility, and robustness for a point-of-use (PoU) setting yielding LoD levels of 0.001 ng/ml for atrazine and 1 ng/ml for glyphosate. Also, the ML model integration resulted in an accurate prediction rate of ≈80% in real soil samples. Therefore, a universal pesticide screening analytical device is designed, fabricated, and tested for pesticide assessment in real soil runoff samples.

## Introduction

There is a major requirement at present to address environmental sustainability in ecological and agricultural practices as highlighted by the United Nations “2030 Agenda for sustainable development” (Arduini et al., 2020). As a result of this proposition, there has been an influx in studies to develop biosensor technologies meant for green living and monitoring different areas in environmental and agri-food sectors. Despite these efforts, one particular vital component of the environment has been probed comparatively much more sparsely, namely, the soil ecosystem that directly and indirectly affects the agricultural health and throughput as well as ecosystem balance (Bullock and Gregory, 2009). One specific problem is that the application of pesticides in crops and other plants often finds its way seeping into soil in large quantities, and there exists a leaching effect at the soil and groundwater sources (Kellogg et al., 2000). The effect caused by the pesticide residues in soil is two-fold: 1. There is a definite relationship between long-term, low-dose exposure of any kind either ingestion, inhalation, or through contact and human health effects such as immune suppression, hormone disruption, diminished intelligence, reproductive abnormalities, and cancer; 2. impact on the environment via surface/groundwater contamination and soil contamination–mediated damage to non-target species–soil biomass, and microorganisms causing decreased crop throughputs and food quality, affecting global food security (Aktar et al., 2009; Lo, 2010; Joko et al., 2017; Gunstone et al., 2021).

The potential for a sensor system that detects in field is given as follows: the levels of pesticide residues in soil runoff is immense and would be beneficial to understand the negative effect of mismanagement and overuse of pesticide agents on food safety and overall quality of life. This field-deployable sensor probe would help promote responsible agricultural practices and curb the overapplication of harmful agents to the soil (Ali et al., 2020). Currently, it is rare to quantify and test for pesticide residues in soil, and even if it is performed, it is done for mostly one analyte, while the field norm in assessing residue levels is reliant on mainly chromatographic techniques (LC-MS/MS, GC-HRMS) with the QuEChERS sample preparation methodology (Silva et al., 2019). Samples in the liquid state requiring complex preprocessing have been tested in different scientific studies and in some cases commercially available test kits utilizing different methods such as capillary electrophoresis (Chang and Liao, 2002), spectrophotometry (Catrinck et al., 2014), and liquid chromatography (Chamkasem and Harmon, 2016) which possess the desired sensitivity and selectivity. A comprehensive summary table has been created, detailing output metrics between different analytical methods versus the sensor performance as a function of processing steps involved, determination/detection time, and limit of detection (LoD), as given in the supplementary section (Supplementary Table S1). However, as mentioned previously, the cost of using these techniques is large overhead in terms of sample collection and transfer, need for sample processing, complex machinery, and reagents as well as the added costs associated with all these steps. Therefore, detection of these pesticides in trace levels in real time in the soil matrix (runoff water) is highly desirable with minimal or no pre-sample processing step involved.

Electroanalytical chemistry proves to be a viable choice of application for such a sensor to track pesticides in soil samples due to its feasibility for *in situ* analysis used as well as solving for the ASSURED criteria as given by the World Health Organization (WHO), wherein it denotes Affordable, Sensitive, Specific, User-friendly, Rapid, and robust, Equipment-free, and Deliverable to end-users (Lim and Bonanni, 2020). The proposed system would have to utilize minimally complex sensor modification/functionalization and no preprocessing of samples. By studying the levels of soil contaminant residues at the field site, the sensor acts as a screening instrument for soil pollution levels.

Hence—citing all these factors and requirements—in this work, we evaluate an electroanalytical sensor approach toward rapid, on-demand screening of 2 commonly used pesticides in this proof-of-feasibility study—glyphosate and atrazine in soil runoff which have a half-life around 60 days^1^ and 60–75 days (Hanson et al., 2020) in soil, respectively. The rationale behind testing these 2 particular pesticide groups is that recently with the agricultural and related developments and the introduction of genetically modified plants, the use of the pesticides such as glyphosate and atrazine has increased to a larger extend worldwide (Battaglin et al., 2009). Glyphosate [N-(phosphonomethyl)glycine] is a polar pesticide used to control the plant weeds by inhibiting the synthesis of amino acids required for the growth of the plants. The most commonly being used is genetically modified glyphosate-resistant crops such as corn, soybean, and cotton crops (Duke and Powles, 2008). Similarly, atrazine (2-chloro-4-ethylamino-6-isopropylamino-1,3,5-triazine) is another pesticide which is largely non-polar used for the pre- and post-emergence control of weeds, especially in sugarcane, maize, and sorghum crops (Oliveira et al., 2015). With the intense use of these two most used pesticides, several concerns have been raised due to the possible residue levels in the soil, water, and plants, a potential threat to the environment and human health (Van Bruggen et al., 2018). With the possible link to the human conditions such as the disruption of endocrine hormones, a potential cause of various cancers (Gillezeau et al., 2019), the U.S. Environmental Protection Agency (EPA) has set the maximum acceptable limit (MRL) of these pesticides in drinking water and various food commodities before they are being marketed (Ambrus, 2015).

In our previous work, we have shown a sensing methodology to test water and produce groups for glyphosate (Dhamu and Prasad, 2020; Dhamu et al., 2021) and atrazine (Pichetsurnthorn et al., 2012), respectively, using an affinity-based biosensing mechanism that is highly specific to the target species. This sensor functions based on an affinity biosensing mechanism driven via thiol cross-linker and antibody receptors that holistically behaves as a recognition immunoassay stack that is specific and sensitive to track test pesticide analytes in the parts per billion (ppb) range for glyphosate and parts per trillion (ppt) range for atrazine. Herein, this universal sensor architecture and chemistry is built and optimized to survey atrazine and glyphosate pesticide panel in soil runoff samples. Here, the universal sensor panel refers to the ability to use and deploy the same 2-electrode design, hardware interface, electrochemical mode (electrochemical impedance spectroscopy; EIS), and similar affinity binding stack respective to each target analyte.

Then, this developed sensor is integrated further to create a pesticide-sensing ecosystem using a front-end field-deployable smart device that drives the electrochemical signal from a potentiostat and then performs computational regression modeling on-chip to denote the resultant trace pesticide level output. Therefore, a universal pesticide-screening analytical device is designed and fabricated for pesticide assessment in real soil runoff samples.

## Materials and Methods

All data graphs were plotted and analyzed using GraphPad Prism, with error bars as mean with the standard error of mean (SEM). All electrochemical experiments were performed using the Gamry Reference 600 potentiostat (GAMRY Instruments, United States).

**FIGURE 1:**
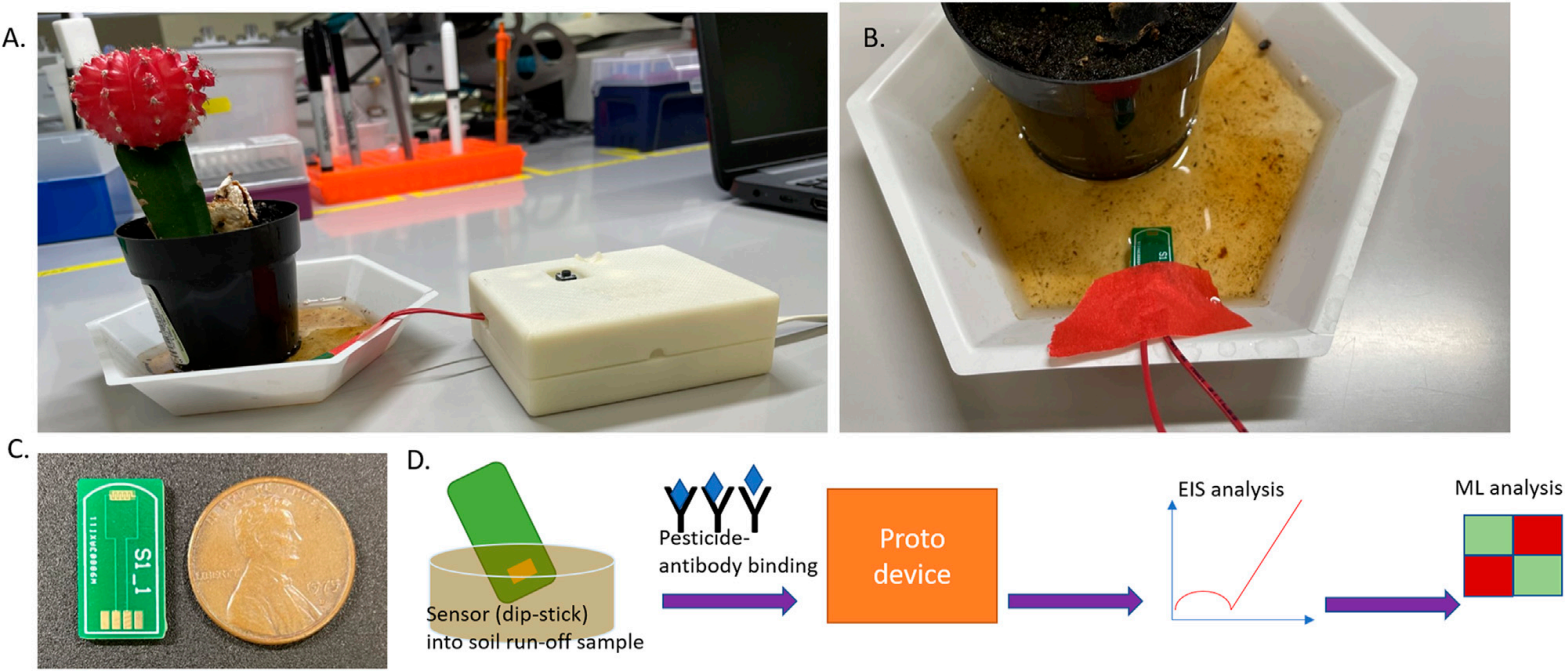
**(A)** Representation of the test setup (from left)- Potted plant filled with garden soil and watered. Soil runoff is collected in the bottom cup (white) and interfaced with the hardware prototype platform simulating in-field deployability and analysis. **(B)** Picture depicting the sensor dipped into the soil runoff sample for measurement. **(C)** USB form factor sensor chip used for pesticide analysis in this work. **(D)** Flow diagram denoting the informational flow regarding the sequence of analysis to track pesticides in runoff: sensor stage (affinity binding chemistry)> device integration> EIS (electrochemical analysis)> machine learning classification.

The sensor design depicted in Figure 1C that was designed and tested in-house was then fabricated on a PCB substrate, manufactured by PCB Way (HK WEIKU Technology Company Limited, China). The fabrication was a single-layer (Top layer) deposition with the conductive layer (copper layer), solder mask, and overlay (silkscreen layer). Here, the conductive layer holds the immersion gold, which is an electroless nickel metal (ENIG) plating technique with a thin layer of gold finish. This provides the electrical connectivity required for the sensor, while the solder mask provides insulation to the rest of the sensor region. The silkscreen layer is added for functionality and depicts sensor chip boundary regions, thereby giving the necessary interfacing capability with the electronic reader (USB-Flash drive design to slot into reader port). The PCB substrate material type used is FR-4 TG-130, with a thickness of 1.6 mm (6/6 mil track/spacing), and the overall dimension of the sensor chip is 17.8 × 9.7 mm.

### Preparation of Soil Runoff Samples

Garden soil used commonly for horticultural use was procured from a commercial source (Home Depot, GA, United States). A standard small cylindrical pot with a hole at the bottom was filled with this soil, and a common cactus was planted which acts as the sample setup for this study. Filtered RO (Reverse Osmosis) grade water was used to water the plant setup, and the excess water (runoff) was collected using a cup holding the pot within it, as shown in Figure 1A. This water was collected and was used as the stock dilution to serially dilute and create the pesticide (antigen) doses with atrazine and glyphosate, respectively. Then 1 mg of the solute (atrazine salt) was mixed into 2 ml of the runoff water and then sonicated for 1 h, following which this concentration of 500 ug/ml was serially diluted down to the test range of 1 pg/ml–1ug/ml. Similarly, with the glyphosate salt, 1 mg was mixed into 1 ml of the soil runoff and mixed. The difference with glyphosate was that the solution forms readily as it is a polar organic molecule which is dissoluble in water even under lower solvent volumes. Then this 1 mg/ml stock concentration of glyphosate soil runoff sample was diluted to the test range between 1 ng/ml and 10 ug/ml.

### Electrochemical Immunoassay Protocol for Affinity Sensing Approach

Phosphate-buffered saline (PBS) (pH 7.4) was used as the solvent and diluent in this set of experiments. To record the binding events at the electrode region between the antibody and pesticide antigen (glyphosate and atrazine), a 10 mV AC bias was applied at the WE and impedance recorded by electrochemical impedance spectroscopy (EIS) to effectively polarize and capture the electrode–electrolyte interfacial effects. Sensor chips were prepared by cleaning with isopropyl alcohol, followed by deionized water. The whole protocol was devised for one individual chip, and each chip was dosed serially from the lowest to the highest dose (ascending order). Experiments were conducted in triplicate, that is, 3 unique sensor chips.

Once the sensor was prepared, 5 μL of cross-linker dithiobis(succinimidyl propionate) (DSP) (Thermo Scientific, United States) made in dimethyl sulfoxide (DMSO) was dispensed onto the gold electrode-sensing region and incubated for 90 min, providing sufficient time for the DSP molecules to be immobilized onto the gold surface. The sensor surface was then coated with 5 μL of glyphosate antibody (chicken polyclonal) solution and atrazine antibody (sheep polyclonal) solution (Fitzgerald Antibodies, United States) of 500 μg/ml and 100 μg/ml, respectively, and allowed to incubate for 30 min undisturbed. Next, 5 μL of superblock (blocking buffer) (Thermo Scientific, United States) was dispensed to the electrode surface and incubated for 10 min to minimize unspecific binding by hydrolyzing the cross-linker functional sites.

The glyphosate antigen (Sigma Aldrich, United States) doses of the range of concentrations required between 1 ng/ml and 10 μg/ml were prepared as described before using soil runoff. Similarly, for the atrazine pesticide (Sigma Aldrich, United States), it was serially dosed in the range of 1 pg/ml–1 μg/ml. Blank soil runoff with no detectable pesticide traces (baseline/negative control) was added to the sensor surface, incubated (10 min), and measured as zero dose (ZD). Then, the fluid was aspirated from the electrode surface, and this procedure was repeated for the next corresponding doses in increasing concentration for the same incubation interval to promote pesticide-antibody binding to take place. EIS measurements were taken after each incubation and dosing step of the immunoassay.

## Results and Discussion

### EIS Methodology for Pesticide Residue Analysis

This section explains in detail the biosensing chemistry that is surveyed using impedimetric analysis that can be captured as a function of pesticide levels in the target sample: soil runoff matrix. Conjugation of the immunoassay stack to specifically recognize the pesticides-glyphosate (GLP) and atrazine (ATR) was implemented on the ENIG immersion-gold finish PCB (FR-4) interdigitated electrode. This stack effectively creates a double layer at the electrode-electrolyte interface that gets modulated due to the presence of increased target analytes in the solution. To explain this concept in detail, we point to the electrical double layer (EDL) structure (Munje et al., 2015; Munje et al., 2017) created when a conductive or semiconducting surface is in contact with a fluid matrix. The EDL consists of a chemically adsorbed layer of charged molecules followed by a layer of oppositely charged species held together via charge attraction (Coulomb forces). What follows is defined as the diffuse layer that can be visualized as ions that move within the fluid under the influence of the applied electrical field and whose strength is proportional to the distance from the contact layer defined formerly.

The detection strategy employed using affinity biosensing is reliant on the following concept: The gold electrode surface is dispensed and conjugated with a thiol cross-linker molecule dithiobis(succinimidyl propionate) (DSP) that binds to the gold layer by thiol–SH linkage, while the opposite end is the NHS ester group end (Xue et al., 2014; Kamakoti et al., 2018). Based on prior characterizations and studies into the design and building of a biosensor for atrazine and glyphosate, it was possible to obtain the optimum antibody parameters for the experimental protocol and determine suitable concentration; an antibody saturation study was conducted, based on results corresponding to a clearly noticeable linear increase in signal values as we increase the dose antibody concentration (Dhamu and Prasad, 2019). It was determined that based on signal saturation at a particular antibody dose, 500 μg/ml for glyphosate and 100 μg/ml for atrazine was the optimum concentration of antibody required with respect to this IDE sensor system. The specific antibody based on the sought-after target group is then functionalized onto the immunoassay that is confirmed by the breakage of the CO–NHS bond in the cross-linker layer and formation of amide bonds I and II, reiterating the binding of the antibody to the DSP cross-linker and thereby the sensor surface itself as understood and proven via Fourier transform infrared (FTIR) spectroscopy in the scientific literature that is relevant here to characterize standard antibody stack conjugation (Upasham et al., 2018).

After this stage, the sensing capture biochemistry is now ready for binding with the target species (pesticide groups GLP and ATR). These interactions are captured via capacitive modulations in the EDL using impedance analysis with EIS that is well-suited for studying and modeling complex systems as ours, which in this case is runoff from field soils.

### Electrochemical Impedance Spectroscopy Enabled Pesticide Sensing

After confirmation of immunoassay functionalization on the gold sensor surface, pesticide levels in aqueous systems were determined using non-faradaic EIS. The non-faradaic method implies the ability to detect target analytes without the use of a redox tag or label. This enables the capability of the sensor to thereby be preprocessing-free and hence does not require additional reagents to act as indicators.

As described before, the changes to the analyte under test are observed as a function of capacitive modulations to the EDL. Therefore, non-faradaic EIS is best suited for this role as it is a thorough method to map the subtle chemistry effects at the electrode–electrolyte interface. So, it is possible to obtain high sensitivity by employing this interfacial probing mode (EIS). To explain this phenomenon in detail, the following is considered: When an AC voltage of 10 mV is applied to the electrode, it results in the perturbation of the solid–liquid interface. Furthermore, causing a capacitance (dielectric) modulation in the double-layer structure due to binding between the antibody and the pesticide molecule, that is, EDL capacitance varies with respect to the antigen–antibody binding. The background behind capacitance change in this model has to do with the dielectric permittivity of the system being modulated due to the double-layer structure and length being perturbed.

These variations are surveyed and then subsequently analyzed with the EIS results plotted as Nyquist (Z_real_ vs. Z_img_), Bode phase (Freq vs. Z_ph_), and Bode magnitude (frequency vs. Z_mod_) plots. This whole EDL structure and the impedance parameters associated with the EIS mode can be visualized and modeled using an equivalent electrical circuit called modified Randle’s circuit (Saxena and Srivastava, 2019; Xu et al., 2019; MintahChurcher et al., 2020).

**FIGURE 2:**
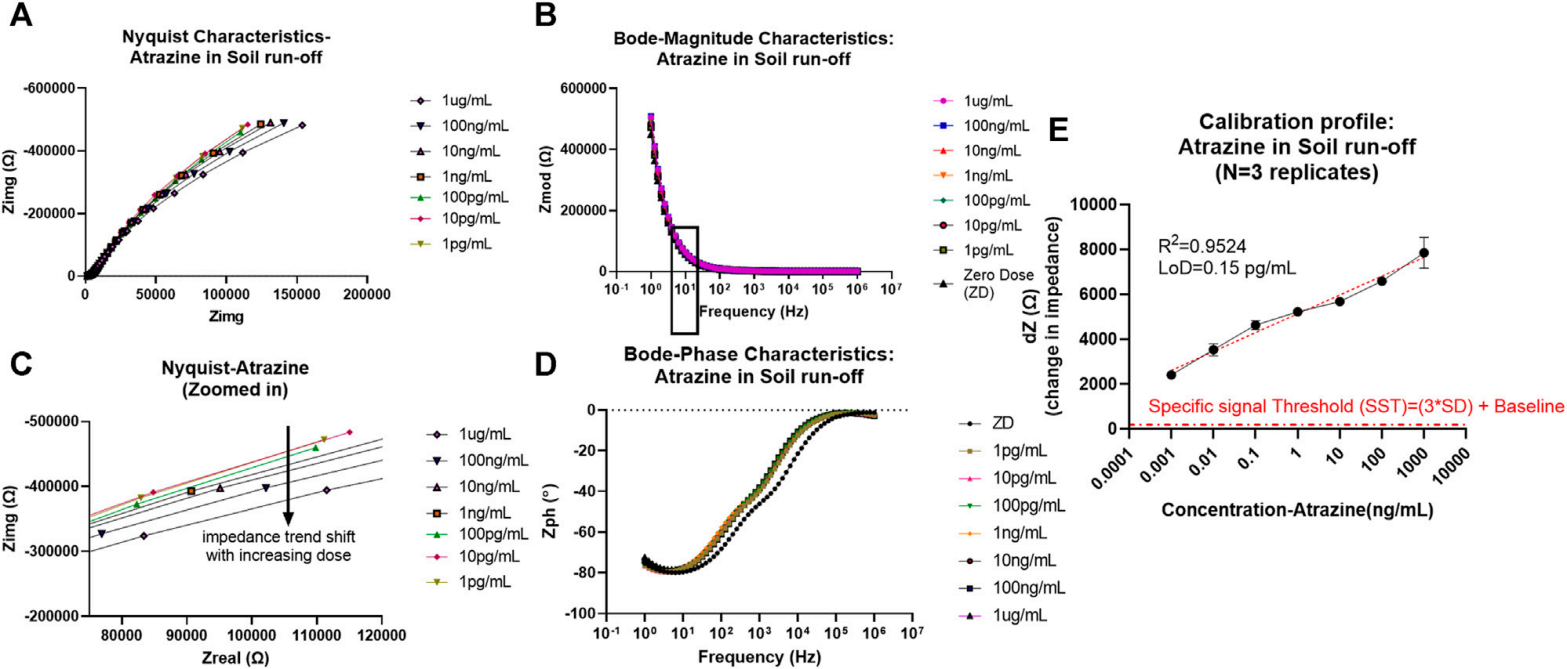
**(A)** Non-faradaic Nyquist characteristic curve depicting the bending of the dose curves with an increase in atrazine concentration toward x-axis (zoomed version of the capacitive region showed in the bottom plot). **(B)** Bode magnitude plot depicting Zmod modulation with dose increase and frequency of interest 10 Hz is marked with a box outline. **(C)** Bode phase plot representation of Zph (angle) trend change from the resistive region at higher frequencies to capacitive domain toward the lower frequencies. **(D)** Calibrated dose–response (CDR) curve with semi-log fit and linearity >0.95 plotted as dZ which is the change in Zmod (for each dose) from baseline signal against the concentration of atrazine in ng/mL.

**FIGURE 3:**
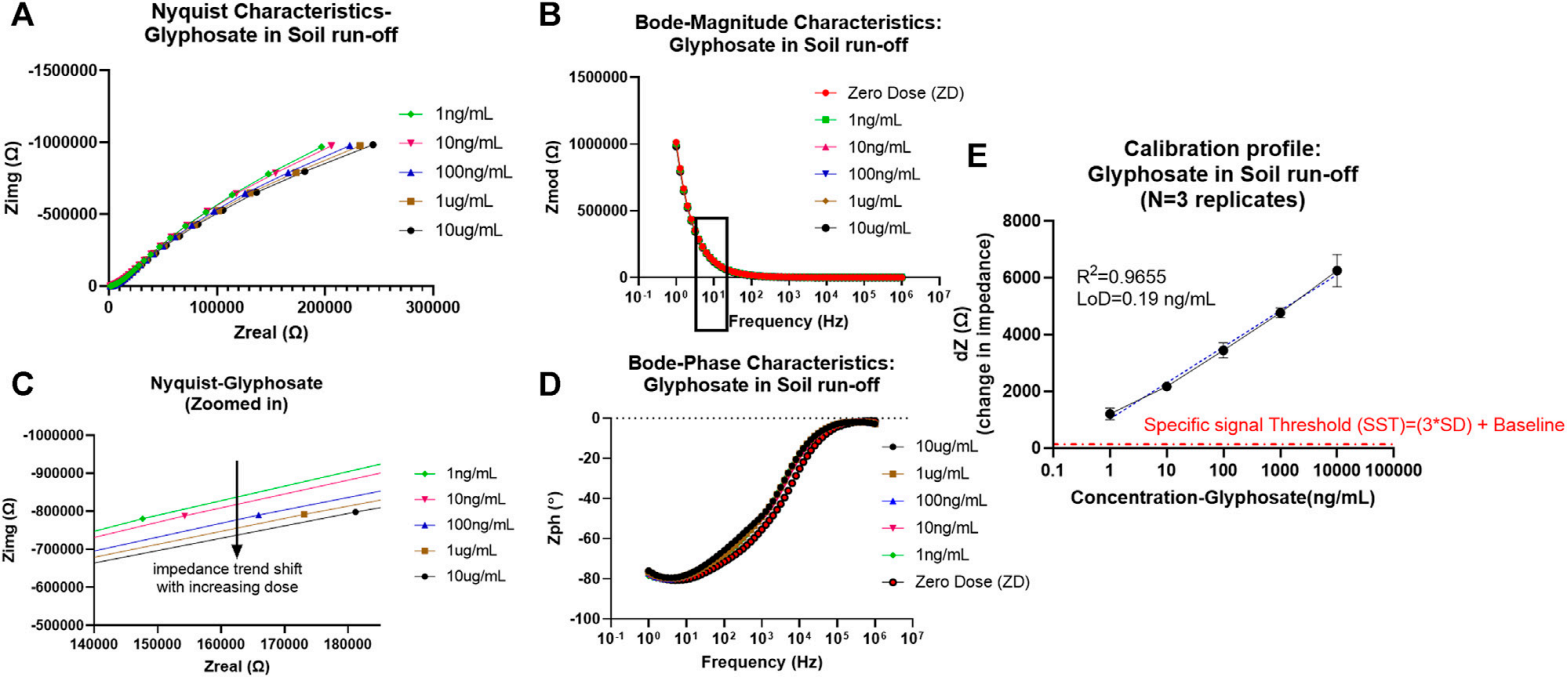
**(A)** Non-faradaic Nyquist characteristic curve depicting the bending of the dose curves with an increase in glyphosate concentration toward x-axis (zoomed version of the capacitive region showed in the bottom plot). **(B)** Bode magnitude plot depicting Zmod modulation with dose increase and frequency of interest 10 Hz is marked with a box outline. **(C)** Bode phase plot representation of Zph (angle) trend change from the resistive region at higher frequencies to capacitive domain toward the lower frequencies. **(D)** Calibrated dose–response (CDR) curve with semi-log fit and linearity >0.95 plotted as dZ which is the change in Zmod (for each dose) from baseline signal against the concentration of glyphosate in ng/mL.

As can be seen from the EIS results later, the absence of charge transfer resistance Rct (infinite) in the Nyquist plot denoted by incomplete semicircles is indicative of non-faradaic analysis (Tanak et al., 2019). Another indicative trend noticed from the Nyquist characteristics is that with increased pesticide doses of glyphosate and atrazine, there is an associated reduction in the radius of curvature in the incomplete semicircles. This is seen as the shift toward the x-axis with an increase in dose, as can be seen in the zoomed-in Nyquist plot in Figure 2 (ATR) and Figure 3 (GLY). One major observation made was that due to the presence of bulk molecules in the soil and water as such, the time needed for binding was increased to 10 min.

In addition to this, Bode plots shown for atrazine and glyphosate is representative of the antigen–antibody binding. The magnitude plot shows that with more binding relative to increases in the dose of pesticide, there is a correlated Zmod modulation, while conversely, in the case of the phase plot, there is a change in phase angle values from more negative values (close to −90) corresponding to more capacitive behavior toward zero phase values, indicative of more resistive nature as there are more antigen–antibody interactions.

The binding between the antibody and antigen drives the impedance modulations in this strategy. Probing further, we understand from utilizing EIS mapping at the electrode–electrolyte interface that the concentration of the analyte molecules drives the degree of binding between the antibody and antigen, which translates to the quantitative correlation and modulation in terms of the analyte-to-signal ratio, especially when looking at Randle’s equivalent circuit, the Cdl (double-layer capacitance) or the CPE (pseudo-capacitance) components referring to the capacitive elements that drive this signal as a function of binding interactions that are translated to concentration-dependent responses (Munje et al., 2015).

Randle’s equivalent circuit here refers to the electrical component model that is used to mimic the chemical stack at the electrode–electrolyte layer. This is previously simulated with the experimental results to fit and compare the values of the electrical components to determine the parameter that drives the molecular level reactions (Dhamu et al., 2021).

Thereby, EIS analysis was used to map the binding effects between the antigen and antibody occurring at the electrode interface while neglecting other bulk effects and non-specific binding effects that contribute to electrochemical noise. To model and extract sensing performance of the system, the Zmod values were utilized at 10 Hz (capacitive binding dominant region), and the calibration curve was plotted each for atrazine and glyphosate and is explained later. To better visualize the change in signal in the Bode magnitude curves for each concentration of pesticide analyte, the graph was zoomed in to the 10 Hz region to better visualize the trend in Zmod shift with an increasing dose for both atrazine and glyphosate, as depicted in Figure S2.

### Atrazine

From the calibrated dose–response (CDR) curve, the sensor response was measured/calibrated against the baseline or zero dose signal value. Thus, the resultant plot was between the dZ (change in impedance from baseline) signal on the y-axis versus the dose of atrazine (ng/ml) on the x-axis. The semi-log curve fitting was used to plot the sensor dose response and extract the parameters of limit of detection (LoD, measured based on a specific signal threshold (SST) as 3*standard deviation (SD) of baseline + baseline level) value of 0.15 pg/ml and sensitivity being −838.6 Ω/log(ppb). Also, the operable limit of quantification (LoQ) for confident tracking and sensor functionality was determined by experimental characteristics to be 1 pg/ml. The values of concentrations chosen in this study for the experimental cycle are in log scale. So, semi-log (x-axis concentration in log scale and y-axis signal in linear scale) fitting was best suited for our analysis, giving a desirable linearity factor of R^2^ = 0.9524 (>0.95). EIS was consequently down-selected as the primary probing mode after comprehensive analysis to track pesticide levels in samples using an affinity biosensing approach. Additionally, the SST also showed that the signal-to-noise ratio (SNR) of this system was appreciable to keep all unspecific noise below its threshold level, and all the desirable concentration-dependent signals are captured efficiently.

### Glyphosate

Similarly, the CDR curve was determined and plotted for glyphosate in an equivalent manner. The system showed improved performance from the atrazine case with linearity R^2^ = 0.9655, partly attributed to its polar nature and thereby better binding and diffusion into the EDL. Operable LoQ obtained was 1 ng/ml (1ppb), which is sufficient based on MRL levels for most agricultural cases, while the LoD was calculated to be 0.19 ng/ml (ppb). The LoD was determined based on the SST in the same manner, as described before for atrazine (3*SD of baseline + baseline impedance value). Additionally, the sensitivity score also got better with 1,271 Ω/log(ppb).

It is possible for the system to capture a dynamic range of 1 pg/ml–1 μg/ml (atrazine) which is in the ppt range and 1 ng/ml–10 ug/ml (glyphosate) therein in the ppb range making it a sensitive, robust, and viable sensor. Additionally, the stability of this method was surveyed using EIS mode and results are denoted using an error bar with ± standard error of mean (SEM).

### Point-of-Use (PoU) Testing and Feasibility via Electroanalytical Sensing Device

The emstat pico module (PalmSens BV, Netherlands) was utilized as the core element of the circuit and interfaced for I/O operations to the MKR zero (Arduino, Somerville, MA, United States) microcontroller system. Together this constitutes the prototype device package used within this study for potted-plant soil runoff experiments. The software interface in use for data collection and subsequent analysis with this device is the PSTrace software under the PalmSens BV banner. A wired connector was used to interface the USB form factor-IDE sensor with the device as shown in Figure 1. This hardware platform was put together into a 3D printed housing construct and interfaced with a tablet/laptop computer for GUI analysis and experimental data collection by running EIS with similar settings as the lab instrument explained previously in the methods section (10 mV AC bias with a frequency sweep between 50000 and 5 Hz).

**FIGURE 4:**
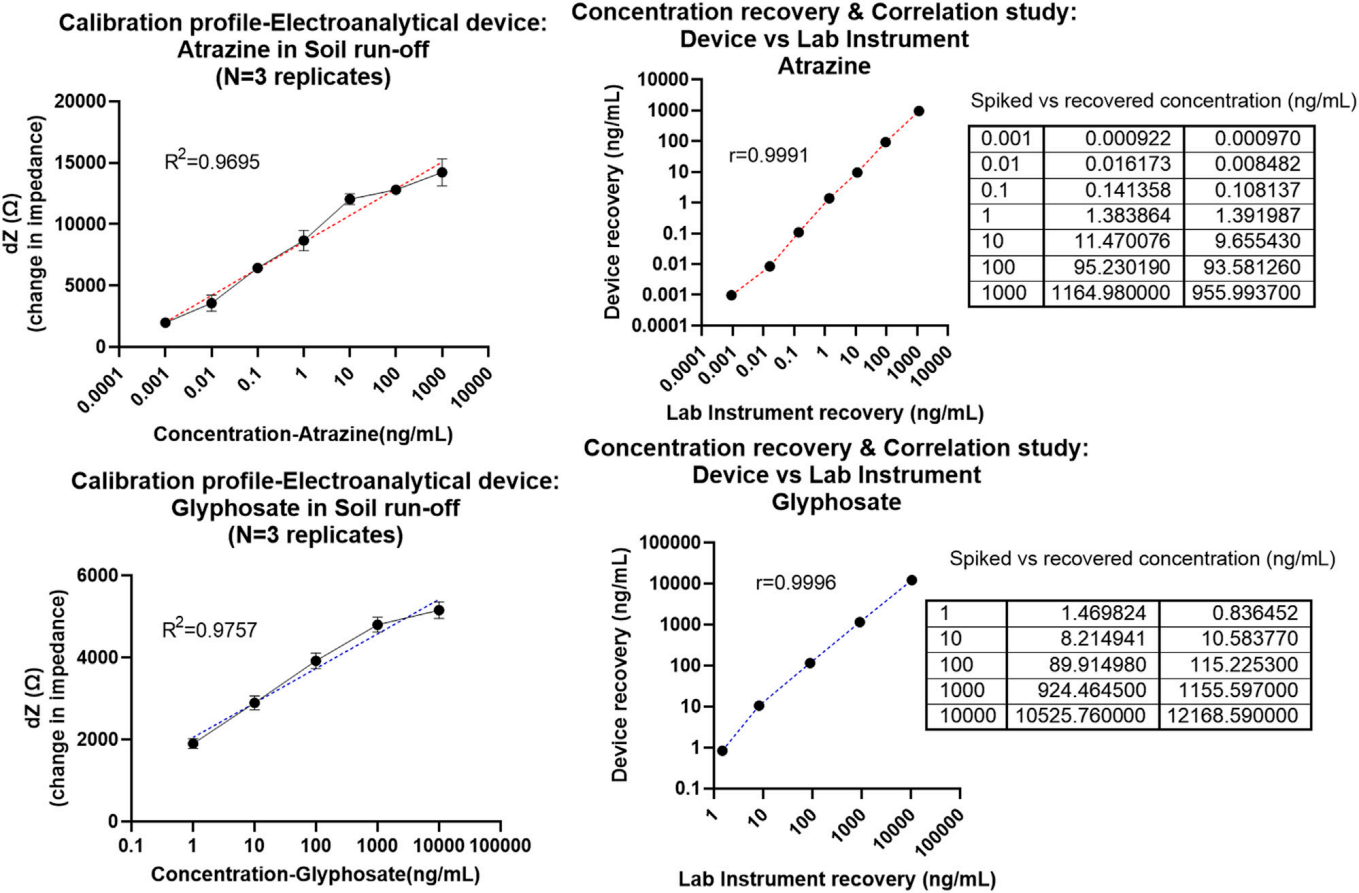
**(A)** Calibrated dose–response (CDR) curve with semi-log fit and linearity >0.95 plotted as dZ which is the change in Zmod (for each dose) from baseline signal against the concentration of atrazine in ng/mL measured using the prototype device. **(B)** Correlation plot given as atrazine concentration recovered using the lab instrument on the x-axis (i.e., performance of benchtop instrument reference) vs. concentration recovered using a proto device on the y-axis (test performance of electroanalytical PoU system). The table on the right shows actual vs. recovered concentrations behavior for laboratory instrument (column 2) and proto device (column 3). **(C)** Calibrated dose–response (CDR) curve with semi-log fit and linearity >0.95 plotted as dZ which is the change in Zmod (for each dose) from baseline signal against the concentration of glyphosate in ng/mL measured using the prototype device. **(D)** Correlation plot given as glyphosate concentration recovered using the laboratory instrument on the x-axis (i.e., the performance of benchtop instrument reference) vs. concentration recovered using a proto device on the y-axis (test performance of electroanalytical PoU system). The table on the right shows actual vs. recovered concentrations behavior for laboratory instrument (column 2) and proto device (column 3).

Similar to the previous section, calibration response curves extracted from the Bode magnitude (Zmod) plot at 10 Hz were obtained for the atrazine and glyphosate analytes, as shown in Figure 4. All measurements and tests in this phase of the study were conducted in triplicates, that is, *N* = 3 independent sensor chips with 3 loops each acting as internal replicates.

The impedimetric response plotted as signal change (dZ) from baseline is fitted as a semi-log non-linear curve in Figure 4 depicting a linearity coefficient R^2^ of 0.9695 for atrazine (Figure 4A) and 0.9757 as in the case of glyphosate (Figure 4C). It was notable that the analytical device system was able to capture the whole dynamic range of 0.001–1,000 ng/ml (atrazine) and 1–10,000 ng/ml (glyphosate) with the dotted line curves representative of the 95% CI line fit which lies on top of the dose–response curve. This is indicative of the feasibility and robustness of this sensor ecosystem. It was observed from the non-overlapping error bars that the PoU device can reliably distinguish between each dose and its subsequent concentration in a thorough manner. Furthermore, statistical ANOVA test of *p* < 0.05 (95% CI) yielded and cemented the hypothesis that different concentration doses are differentiable from each other with statistical significance. The system was able to effectively read concentrations as low as 0.001 ng/ml (LOD-atrazine) and 1 ng/ml (LOD-glyphosate) with respect to a 3*SD-specific signal threshold (SST) calculation.

Next, to determine the applicability and performance metrics of this PoU device, the dose–response results obtained from the laboratory reference instrument were compared to that evaluated using the fabricated prototype device. Herein, the laboratory instrument which was a Gamry potentiostat system is taken as the standard potentiostat to which the proto device output is compared. By applying the calibrated line equation model [y = m*ln (x) + c], the output signal (y-axis in CDR curve) was used to reverse calculate the concentration (x-axis on CDR curve) and “m” denotes the slope of the line and “c” represents the intercept value. Thus, the signal from the experiment is used to obtain the recovered concentrations against the standard curve fit. From this step, we obtain 2 key performance metrics: First, Pearson correlation analysis performed between the results from two devices (methods); laboratory instrument vs device is used to validate the feasibility of system toward in-field deployability. A linear matching between the recovered concentration dataset (plotted in y-axis as device results) and that of the laboratory instrument results (plotted in x-axis) is compared by determining Pearson’s correlation coefficient “r” of 0.9991 in the case of atrazine and 0.9996 for glyphosate. Therefore, the proposed system showed excellent correlation with the laboratory instrument sensor response data with a high r-value close to 1 (ideal).

Second, the efficacy of the sensor system in translating signal values to pesticide contamination levels was determined using the spike and recovery table seen on the right side of Figures 4B,D. Even with a complex system like the soil, the system was able to capture the pesticide levels (recovered concentration values) within ± 20% error of the actual concentration present in the sample. This makes the proposed platform viable for in-field testing and broad-scale use.

### Machine Learning Classifier Integration

The pesticide contamination machine learning (ML) classification model and application GUI (for use in Windows) were implemented using MATLAB (Natick, MA, United States)—Classification Learner application to create the training model and then perform subsequent testing. The model for the training dataset was tested using 5-fold cross-validation (Lakshmi and Rao, 2019), and the algorithm for ML processing was based on bagged trees logic which is a commonly used method for classification purposes (Al-Barazanchi et al., 2017; Widasari et al., 2020).

The utility of the PoU platform is enhanced by the integration of the classifier algorithm and is used to segregate sample pesticide contamination into 3 output classes:

Atrazine.

 1) 0–0.1 ng/ml (low)
 2) 0.1–100 ng/ml (mid)
 3) >100 ng/ml (high)

Glyphosate.

 1) 0–10 ng/ml (low)
 2) 10–100 ng/ml (mid)
 3) >100 ng/ml (high)

Experimental output data collected from the proto device platform contain column-wise information as follows: Zreal, Zimg, Zph, Zmod, frequency, and dose within each measurement cycle. The novelty in this proposed classifier is the ability to obtain ML classification output into the aforementioned 3 classes by exporting this output data file directly into the computational system without any need for preprocessing or data consolidation. The factors, namely, frequency, Zreal, Zing, and Zmod, together were seen to be optimized predictors for the model with the response as tertiary classes based on observations as described before: low, mid, and high contamination classes.

**FIGURE 5:**
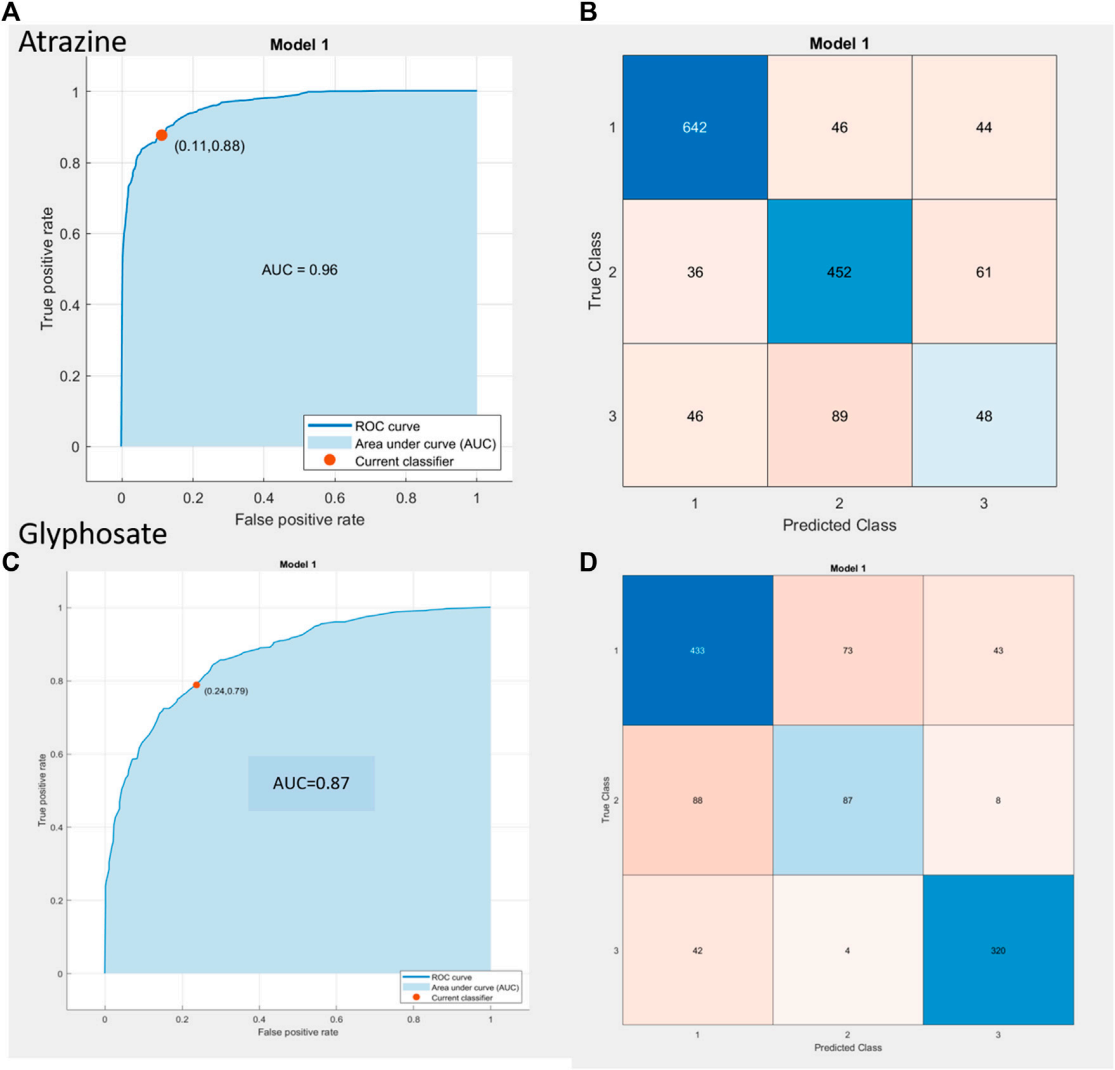
**(A,C)** ROC curve results for atrazine **(A,B)** and glyphosate **(C,D)** inclusive of AUC value, TPR, and FPR value (represented as the red dot with values in parenthesis). **(B,D)** Confusion matrix depiction to determine true class vs predicted class behavior for ATR **(A,B)** and GLY **(C,D)**, respectively.

The data input is from *N* = 3 independent sensor replicates with 3 loops each for 8 concentration doses for atrazine and 6 concentration doses for glyphosate yielded 1,464 and 1,098 unique observations/sample points for the two pesticide compounds, respectively. This is the input used to build and test the ML model whose results are depicted in Figure 5 as a receiver operating characteristic (ROC) curve plotted between the true-positive rate (TPR) on the x-axis and false-positive rate (FPR) on the y-axis. The choice was finalized following the review of output parameters such as accuracy in % and the ROC curve characteristics (i.e., maximum TPR and minimal FPR). From the ROC curve results for atrazine, it can be seen that the TPR/FPR ratio is sufficient taking into account the complex nature of soil along with the overall accuracy being 79.4% and area under the curve (AUC) value of 0.96. Additionally, on the right side of the figure, the confusion matrix is seen with the predicted class on x-axis versus the true class on y-axis. Classes 1, 2, and 3 refer to the low, mid, and high contamination levels, and from the heat map for atrazine, it is seen that the error is more prominent at the mid-high contamination level borders, while there is a good degree of confidence at the lower concentration ranges. For the case of glyphosate, the ROC curve yields the performance metrics of 76.5% overall accuracy and AUC value of 0.87; the error for glyphosate was concentrated for the most part in the mid-level contamination region. It is the assumption of the authors that adding more data points from different soil types and an additional number of sensor results would cause the model to auto-resolve further and optimize the performance to yield even better results.

## Conclusion

In this study, we have successfully put forward a sensing ecosystem to track a pesticide panel of different types—atrazine (non-polar) and glyphosate (polar)—using the same universal sensor design and setup. This solution holds the potential to be deployed *in situ* as a first response to screening and solving for soil pollution, food quality understanding, and thereby boosting food security.

It was possible to depict sensor performances in the range of parts-per-trillion detection limits for atrazine and parts-per-billion in the case of glyphosate with runoff from actual real field soil samples. A feasibility study was undertaken to cement the field deployability aspect by designing an electroanalytical device to run EIS on the samples in a portable setting and thereby obtaining the trace residue levels in an on-demand manner. The performance from the electroanalytical system was correlated to that obtained from the laboratory-grade benchtop instrument indicating the stability and robustness of the proposed platform for agricultural field tests in wet farms that grow paddy (rice), etc.

## Data Availability

The raw data supporting the conclusion of this article will be made available by the authors, without undue reservation.

## Footnotes

1 Report-Technical factsheet on glyphosate by United States Environmental protection agency (US-EPA)
