# Supplementary material for: Targeted On-Demand Screening of Pesticide Panel in Soil Runoff
Source: Front Chem. 2021 Nov 30;9:782252. doi: 10.3389/fchem.2021.782252 (PMC8670307; doi:10.3389/fchem.2021.782252)
Supplement: Supplementary file 1 [file DataSheet1.docx]

Targeted On-demand screening of pesticide panel in soil run-off

Vikram Narayanan Dhamu^a^, Suhashine Sukumar^a^, Crisvin Sajee Kadambathil^a^, Sriram Muthukumar^b^, Shalini Prasad^a^*

*^a^ Department of Bioengineering, Biomedical Microdevices and Nanotechnology Laboratory, University of Texas at Dallas, TX, Richardson, USA*

*^b^ EnLiSense LLC, TX, Allen, USA*

**Corresponding Author:*

*Dr.Shalini Prasad,*

*Shalini.Prasad@utdallas.edu*

*Department of Bioengineering, The University of Texas at Dallas, 800 W Campbell Rd, Richardson, 75080, TX, United States*

Supplementary data:


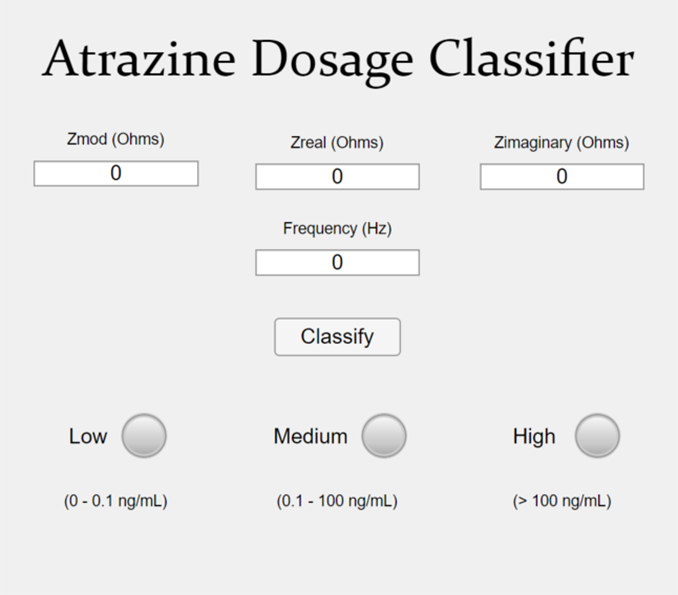


Figure S1. Front-end GUI design of the app for ML classification of Atrazine pesticide levels in soil run-off (segregated into 3 output classes-Low, Medium and High)


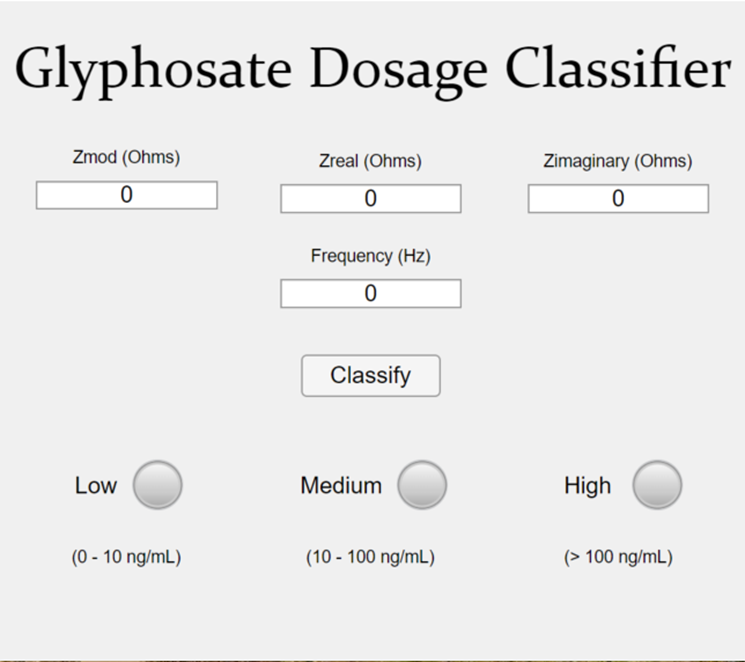


Figure S1. Front-end GUI design of the app for ML classification of Glyphosate pesticide levels in soil run-off (segregated into 3 output classes-Low, Medium and High)


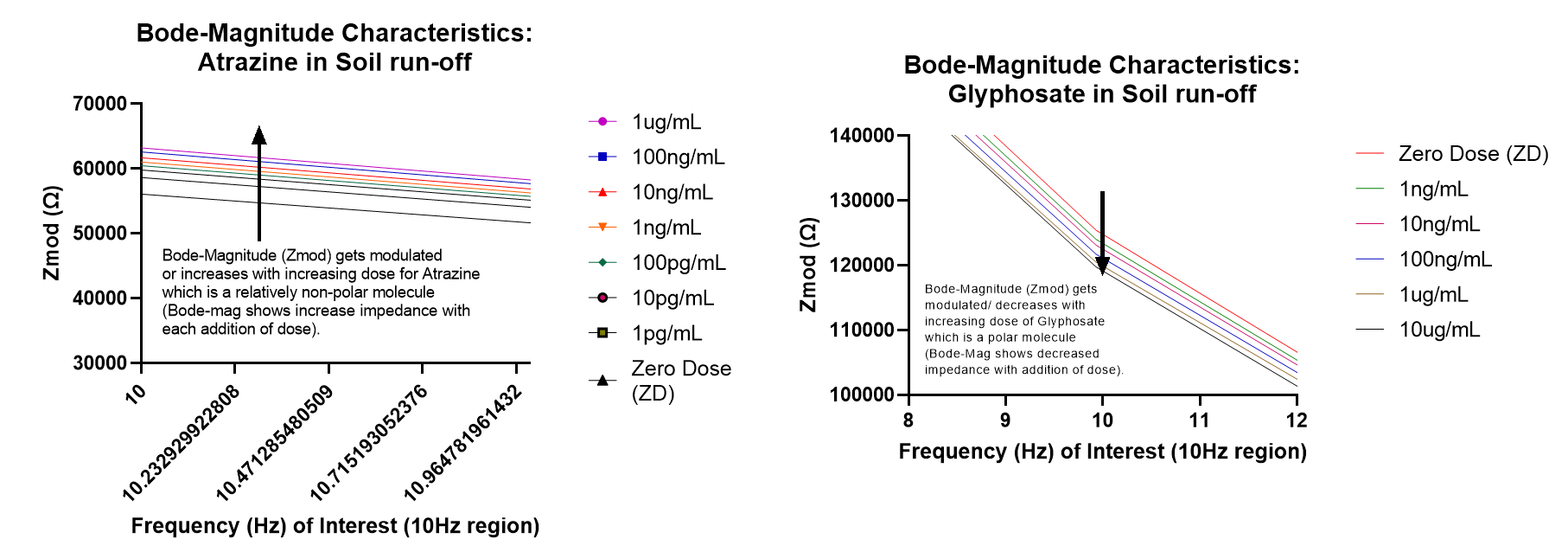


Figure S2. Bode-Magnitude plot depicting Zmod modulation with dose increase zoomed into frequency of interest (10Hz) depicting Zmod curve shift trend with dose increase (left) increasing Zmod value for Atrazine and (right) decrease in Zmod value for Glyphosate.

| **Analytical method** | **Material/method system** | **Sample system** | **Detection Time including extraction/ derivatization (if applicable)** | **Limit of Detection (LoD)** | **Reference** |
| --- | --- | --- | --- | --- | --- |
| (GC) Gas Chromatography-NP detector | Derivatizing agent- trifluoroacetic anhydride + trifluoroethanol | Glyphosate in soil | > 2Hr | 9pg/g of sample | (Hu et al. 2008) |
| (LC-MS) Liquid Chromatography-Mass Spectrometry | Derivatizing agent- 9-Fluorenylmethylchloroformate | Glyphosate in soil | >15 Hr | 0.05 mg/Kg of sample | (Botero-Coy et al. 2013) |
| UV Spectroscopy | Derivatizing agent- Carbon Disulphide | Glyphosate in soil solution | >24 Hr | 1.1 µg/mL | (Jan et al. 2009) |
| Fluorescence | Escherichia coli phosphonate-binding protein (PhnD) with fluorophore | Glyphosate in Soil solution | >2 Hr | ≈10µM | (Y N’Guetta et al. 2020) |
| Spectroelectrochemical | Nano-ZnO modified gold MWCNTs/SPE | Glyphosate in soil | - | 3µM | (Teofilo et al. 2004) |
| Enzyme Inhibition biosensor | Enzyme tyrosinase from mushrooms (Tyr) with MWCNT electrode modification | Atrazine in drinking water | 20 min | 0.3 ppm | (Tortolini et al. 2015) |
| Gas Chromatography–Mass Spectrometry | bubble-in-drop single-drop microextraction | Atrazine in soil (Extracted solution phase) | ≈1 Hr | 0.01 ng/mL | (Williams et al. 2014) |
| ELISA | Polyclonal Antibody (PAb) | Atrazine in soil | >2 Hours | 0.025 µg/L | (Deng et al. 1999) |
| This work* | Standard Antibody Conjugation to sensor (No additional modification to electrode) | Glyphosate in soil run-off | 10 min | 0.19 ng/mL in Lab system and 1 ng/mL in test portable device. | |
|  |  | Atrazine in Soil Run-off |  | 0.15 pg/mL in Lab system and 1pg/mL in test portable device. | |

Table S1. Comparative table describing different approaches to pesticide detection in soil samples with information on material parameters, detection time and detection limit for each method.

Additional NOTE: Based on literature survey and studying different methods and approaches for pesticide detection in soil samples- it was seen that most methods have a requirement for extraction and derivatization processes prior to be able to detect pesticides in sample. Further, the time required to perform these steps in addition to complexity of the process is one notion to consider while evaluating different techniques.

The proposed sensor does not require major pre-processing of sample and also independent of complex electrode modification for sensitivity increase.
